# Aging Uncouples Heritability and Expression-QTL in *Caenorhabditis elegans*

**DOI:** 10.1534/g3.112.002212

**Published:** 2012-05-01

**Authors:** Ana Viñuela, L. Basten Snoek, Joost A. G. Riksen, Jan E. Kammenga

**Affiliations:** Laboratory of Nematology, Wageningen University, Wageningen, The Netherlands

**Keywords:** life span, aging, linkage, longevity

## Abstract

The number and distribution of gene expression QTL (eQTL) represent the genetic architecture of many complex traits, including common human diseases. We previously reported that the heritable eQTL patterns are highly dynamic with age in an N2 × CB4856 recombinant inbred population of the nematode *Caenorhabditis elegans*. In particular, we showed that the number of eQTL decreased with age. Here, we investigated the reason for this decrease by combining gene expression profiles at three ages in the wild types N2 and CB4856 with the reported expression profiles of the RIL population. We determined heritability and transgression (when gene expression levels in the RILs are more extreme than the parents) and investigated their relation with eQTL changes with age. Transgressive segregation was widespread but depended on physiological age. The percentage of genes with an eQTL increased with a higher heritability in young worms. However, for old worms this percentage hardly increased. Using a single marker approach, we found that almost 20% of genes with heritability >0.9 had an eQTL in developing worms. Surprisingly, only 10% was found in old worms. Using a multimarker approach, this percentage increased to almost 30% for both age groups. Comparison of the single marker to a multiple marker eQTL mapping indicated that heritable regulation of gene expression becomes more polygenic in aging worms due to multiple loci and possible epistatic interactions. We conclude that linkage studies should account for the relation between increased polygenic regulation and diminished effects at older ages.

Most complex traits, including many common human diseases, are heritable, meaning that part of the phenotypic variation is associated with genotypic variation. Theoretically, heritability values range from 0 (*i.e.* variation in the trait is not determined by genetic factors) to 1 (variation in the trait is completely determined by genetic factors). So far, most traits have shown heritability values between 0.2 and 0.8. A key challenge is to identify the genes that explain this heritability by linking phenotypic variation to polymorphic genomic regions in genotyped recombinant inbred lines [quantitative trait loci (QTL) mapping]. High-throughput genome-wide gene expression profiling has tremendously increased the power of QTL mapping with the goal to link (small effect) genes or loci to complex traits. Variation in gene transcript abundance among individuals is heritable in genetically segregating populations (Brem *et al.* 2002; Li *et al.* 2006), which allows for the genetic mapping of gene expression and detection of expression quantitative trait loci (eQTL). eQTL are polymorphic genomic regions associated with individual variation in transcript abundance. They can be *cis*- or *trans*-acting, reflecting local and distant regulation of gene expression, respectively, and they have been studied intensively in an increasing number of species, like humans, yeast, plants, rats, mice, and worms (Li *et al.* 2006, 2010; Brem *et al.* 2002; Schadt *et al.* 2003; Petretto *et al.* 2006; Keurentjes *et al.* 2007; Farber *et al.* 2009; Viñuela *et al.* 2010a). The detection of eQTL provides a powerful means to construct gene regulatory networks underlying many different phenotypes, ranging from life-history traits to complex diseases (Cookson *et al.* 2009). For instance Derry *et al.* (2010) identified eQTL and constructed networks driving cardiovascular and metabolic phenotypes in mouse recombinants. Also the integration of molecular phenotypes, such as gene and protein expression levels in combination with eQTL, can be used to aid the reconstruction of these pathways and genes (Fu *et al.* 2009; Terpstra *et al.* 2010). Next to these studies, evidence is accumulating that single nucleotide polymorphisms (SNP) associated with complex traits in genome-wide association studies (GWAS) are often enriched with eQTL. These enrichments are robust across a range of thresholds for establishing eQTL and a wide range of complex human phenotypes (Nicolae *et al.* 2010). Gamazon *et al.* (2010) found that chemotherapeutic drug susceptibility–associated SNPs are more likely to be eQTL and to be associated with the transcriptional expression level of many genes as potential master regulators, compared with a random set of SNPs found in association studies.

At the moment, however, it is still unclear to what extent the detection of eQTL is determined by the heritability of gene expression. Petretto *et al.* (2006) studied the relationship between heritability and eQTL across different tissues using a population of rat recombinant inbred strains. They reported that the proportion of heritable expression traits was similar in all tissues but concluded that heritability alone was not a reliable predictor of whether an eQTL will be detected. Recently, we reported that age influences the distribution of eQTL and that the temporal dynamics of regulatory loci are heritable in the nematode worm *Caenorhabditis elegans* (Viñuela *et al.* 2010a). But so far, the relationship between eQTL and heritability of transcript abundance in aging organisms has not been elucidated.

Here, we used *C. elegans* to address this question. We estimated the heritability for thousands of transcript phenotypes throughout the whole life of *C. elegans* recombinant inbred lines (RIL) based on data obtained from a previous study (Viñuela *et al.* 2010a) in combination with new transcriptomic data of their parental strains (N2 and CB4856). Both the measurements on parental strains and RILs were conducted at the same time to prevent any batch effects. We first compared genome-wide differentially expressed genes between N2 and CB4856 over the worm’s lifespan, identifying age-, genotype-, and age-by-genotype–affected transcripts. Then we studied transgressive segregation of gene expression as a descriptor of the genetic architecture of gene expression in aging worms. Transgressive segregation in the RILs implies that an allelic combination produced higher or lower expression levels in a specific group of RILs than in either parent. After that, we compared mapped eQTL and age-by-genotype eQTL to the parental expression data. Using transcriptome data of both the RILs and the parental strains, we investigated how heritability of genome-wide gene expression changes with age and to what extent this affected the detection of eQTL in aging *C. elegans*.

Our results are relevant for understanding the biology of aging and longevity. The analysis of transcript abundance in RILs and parental strains indicates that gene expression regulation becomes more polygenic with age and that expression levels of genes involved in damage repair and energy distribution remain strongly regulated at old ages.

## Materials and Methods

The experiments on the parental strains CB4856 and N2 were conducted at the same time with the RIL study as reported in Viñuela *et al.* (2010a). These data were used to estimate heritability of gene expression (see below). In this study, we used a set of 36 RILs, genotyped with 121 evenly spaced single nucleotide polymorphism (SNP) markers, derived from *C. elegans* wild types N2 and CB4856 (Li *et al.* 2006; Kammenga *et al.* 2007). We measured genome-wide gene expression using microarrays from the RILs reared at 24° at three different ages: young worms (t1), age 40 hr; reproductive worms (t2), age 96 hr; and old worms (t3), age 214 hr.

### *C. elegans* culturing

For the wild type parental strains, we followed the same protocol as in Viñuela *et al.* (2010a). *C. elegans* strains N2 and CB4856 were cultured on standard nematode growth medium (NGM) with *E. coli* OP50 as food source and a constant temperature of 24°. Populations were started with nonmated hermaphrodites and screened regularly to remove any occurring males. Reproductive nematodes from both wild types were bleached (0.5 M NaOH, 1% hypochlorite) to collect age-synchronized eggs (Emmons *et al.* 1979), which were inoculated (t0) in 9 cm petri dishes. After 40 hr (t1), nematodes in late L4 stage from 6 dishes were collected as one sample, frozen in liquid nitrogen, and stored at −80° until RNA extraction. The remaining 18 dishes were kept in culture until hour 41, when the nematodes were transferred to fresh NGM dishes (with *E. coli* OP50) treated with 0.05–0.01 mg/ml of FUDR (fluorodeoxyuridine) to avoid egg hatching. After 30 hr, the nematodes with FUDR were transferred to fresh dishes (without FUDR) to prevent starvation and to remove the FUDR. After 23 hr, 96 hr of total culture time (t2), nematodes from 6 dishes were collected and frozen in liquid nitrogen prior to RNA extraction. The remaining 12 dishes were kept at constant temperature until 214 hr of culture (t3), when they were harvested and frozen in liquid nitrogen. All the dishes were visually inspected before harvest. Any population with infection, more than one generation (reproduction), or starving nematodes (lacking bacteria) were discarded.

### Microarray experiments

RNA from nematodes was extracted following the Trizol method, followed by the RNeasy Micro kit (Qiagen, Valencia, CA) to clean up the samples. Labeled cDNA was produced with an Array 900 HS kit from Genisphere and Superscript II from Invitrogen. The Nucleospin kit (Bioké, Leiden, The Netherlands) was used to clean the cDNA samples to reduce unspecific binding to the arrays. The two colors 60-mers arrays were obtained from Washington University (see also Viñuela *et al.* (2010b, 2011).

N2 *vs.* CB4856 samples were hybridized to each array, with six replicates of t1, t2, and t3 in a dye swap design. The microarrays were hybridized following the Genisphere Array 900 HS protocol. The differential hybridization due to SNP differences between N2 and CB4856 is low in these 60-mers arrays (Li *et al.* 2006). All microarray data have been deposited in Gene Expression Omnibus (GEO) with the common accession number GSE22887.

### Microarray normalization

A PerkinElmer scanner was used to extract raw intensities. R software was use for preprocessing and normalization (www.r-project.org) using the Limma package (Smyth 2005). The Loess method (Smyth and Speed 2003) was used for normalization within arrays, and normalization between arrays was done using the quantile method (Yang and Thorne 2003); both methods are included in the Limma package. The expression data from N2 and CB4856 were normalized together with the RIL expression data (GEO accession number GSE17071).

Outliers were removed prior to normalization. Outliers were considered values lower or higher than two times standard deviation of the mean, per spot per stage. Outliers from N2 and CB4856 arrays were removed prior to normalization using four linear regressions: one per genotype (N2 and CB4856) and per age group (t1–t2, and t2–t3). Each regression fitted gene expression values according to the genotype and the two time points (t1–t2 or t2–t3) and removed values outside the 0.995 confidence interval, one value at a time, recursively. No more than six values were allowed to be removed. Expression values from t2 worms were considered outliers if they were removed in either the developing or aging linear regression.

### Using physiological age to correct for developmental differences across the lines

Because the parental lines, as well as the RILs, differed in physiological age (*i.e.* they differed in their absolute life spans), sampling mRNA at a fixed chronological age would implicitly result in different physiological stages being sampled. Therefore, we analyzed gene expression in relation to physiological age, which we defined for each line as the age at the time of mRNA extraction divided by the mean lifespan of that line. In this way, the age-physiological differences among the lines were taken into account when comparing the gene expression profiles. The data of the mean lifespan for the lines were taken from supplemental Table 1 in Viñuela *et al.* (2010a).

### Gene expression mapping

First, we used a linear model (model 1) to calculate the linkage of each marker (in case of the RILs) or genotype (in case of the parents) with the measured expression levels for each of the time points separately. We used the log2 single channel normalized intensities as a measure for gene expression. The model used for each of the three age groups was: gene expression = marker(effect) + error. In this way, we obtained the genome-wide eQTL profiles for all genes for the three age groups and the differentially expressed genes between genotypes in the parental strains (N2 and CB4856).

Second, to quantify the heritable differences in gene expression that are age dependent, we extended our linear model by analyzing two age groups at once and including the physiological age of the RILs, N2, or CB4856 as an explanatory factor (model 2). The model used for both combinations, t1 (juvenile) and t2 (reproductive), and t2 and t3 (old worms), was as follows: gene expression = marker(effect) + physiological age + interaction(marker × physiological age) + error. The age groups t1 and t2 in one model are referred to as “developing worms”; the age groups t2 and t3 in one model are referred to as “aging worms.” The significance and effect of each marker, physiological age (time), and the interaction between the two were obtained for all genes on the array.

### Gene expression threshold determination

We used a permutation approach to determine the thresholds for the differentially expressed genes in the parents. For model 1, we permuted transcript values and used a genome-wide threshold of –log10 *P*-value = 2, which resembles a false discovery rate (FDR) of 0.0129, 0.0118, and 0.0136 for each of the three time-points, respectively. For model 2, we used 100 permutations to estimate the FDR threshold. Per permutation, genotypes and ages were independently randomly distributed, keeping the among-gene structure intact. Then for each spot (23,232) on the array, model 2 was tested. The obtained *P*-values were used to estimate a threshold for each of the explanatory factors. We also used a genome-wide threshold of –log10 *P*-value = 2, which resembles an FDR of 0.072 and 0.060 for marker and the interaction age-marker for the developing worms and FDR of 0.050 and 0.065 for marker and age-marker for the aging worms. For the physiological age effect, we used a –log10 *P*-value = 8 in developing worms (0.012 FDR) and –log10 *P*-value = 6 (0.032 FDR).

### eQTL threshold determination

We calculated *P*-values from permuted data for eQTL mapping and for genotypic effect in the parents for both models as described above (30 permutations). Then, we calculated the probabilities to find genes that were significantly different between the parental genotypes and that also have an eQTL by chance (joint-FDR). This approach allowed us to relax the threshold for the linkage mapping from –log10 *P*-value = 3.8 (Viñuela *et al.* 2010a). For simplicity, we decided to use a level of significance of –log10 *P*-value = 3 for eQTL mapping and a –log10 *P*-value = 2 for the parents analysis for genotype (eQTL) and genotype-by-age interactions (_g×a_eQTL). Those thresholds resembled the following joint-FDR for model 1: 0.0129 and 0.0136, for genotype and the interaction, respectively. The joint-FDR in model 2 for genotype and the interaction in developing worms was 0.058 and 0.044. In aging worms, the joint-FDR was 0.038 and 0.046, respectively.

### Multiple regulatory elements analysis

To investigate whether genes without an identified eQTL could have one or more eQTL, we used a forward and backward marker selection approach in model 2 described in the previous section. To make this procedure possible, we selected 4 markers per chromosome, obtaining a total of 24 markers. All 24 markers, including paired interactions, physiological age, and the interaction between marker and physiological age, were used as start parameters. The Bayesian information criterion (BIC) was used as a selection method to identify the best fit explaining the origin of the variation per gene expression. We allowed 2000 steps and no more than six explanatory variables. From each analysis, the number of markers and their interactions explaining the variation in gene expression was determined and used in further analysis.

### Transgressive segregation

Transgressive segregation in the RILs implies that the allelic combination produced higher or lower expression levels in a specific group of RILs than in either parent. We identified transgression by comparing the expression levels of each gene in the RIL with the parental gene expression. Transgression to higher or lower expression, or to both extremes was defined for those genes with expression values two times standard deviation of the mean expression of the higher or the lower parent in at least six RILs. The FDR as the threshold for number of transcripts (n = 6) was computed (Keurentjes *et al.* 2007). In the three time-point analysis, a threshold of six transcripts resembles an FDR of 0.049, 0.046, and 0.045. Results were averaged over 100 permutations.

To identify transgressive expression over time, we counted those genes with expression values two times standard deviation of the mean of the higher or the lower parent in both t1 and t2 (developing worms), and in both t2 and t3 (aging worms) in at least six RILs. The FDR for this analysis were 0.0099 and 0.0028. Results were averaged over 100 permutations.

### Heritability

We calculated the heritability of each transcript as H^2^ = (V_RIL_ − V_P_)/ V_RIL_ (Keurentjes *et al.* 2007), where V_RIL_ was the variance among the RILs and V_P_ was the pooled within line variance of the parents. We used the same approach to estimate the heritability of gene expression over time; that is, the heritability patterns of gene expression changes. For each transcript, we calculated the variance among the RILs as the variance due to genotypic effect over time (developing and aging worms); and for the parents, variance over time. In this way, the effect of the interaction between physiological age and genotype on the variance was excluded. For model 2, we found 4.54% and 4.47% transcripts with negative heritability in developing and aging worms, respectively.

To identify highly heritable genes, we used a permutation test as in the previous analysis. Transcript values were randomized prior to calculating permutated heritable values per transcript for developing worms and for aging worms. From the permuted values, we identified the higher heritability value that allowed less than 1% false positive (FDR = 0.01), H^2^ > 0.69 for developing worms and H^2^ > 0.77 for aging worms. Results were averaged from 100 permutations per gene.

### Gene Ontology analysis

Gene Ontology (GO) data and functional domain data were extracted from Wormbase release WB210. GO terms with less than two genes were discarded. Overrepresented groups of GO terms and domains were identified using a hypergeometric test (*P*-value < 0.01). In this way, we analyzed 2109 unique GO terms from 18,312 annotated genes, respectively.

## Results

### Age and genotype affect gene expression in N2 and CB4856

The gene expression levels of the parental genotypes of the N2 × CB4856 RIL population were measured for three age groups: juvenile worms, reproductive worms, and old worms. We identified 11.3% (2151), 9.8% (1868), and 14.1% (2679) genes significantly affected by the genotype for each age group, respectively (supporting information, Table S1). The mean lifespan differed between both strains, ∼16 days for N2 and ∼13 days for CB4856 (Viñuela *et al.* 2010a). Age at maturity, on the other hand, did not differ (Gutteling *et al.* 2007a). In other words, physiological age differed between N2 and CB4856 at the time of RNA harvest, and this difference became more prominent at older ages. To correct for this discrepancy, we treated age differences with a two-time model for developing worms (juvenile and reproductive worms) and aging worms (reproductive and old worms) [*cf*. Viñuela *et al.* (2010a)] (Table S2). Three factors were considered: physiological age, genotype, and their interaction. In this way, we found 15.9% (3694) and 11.3% (2626) of all transcripts to have a significant age effect in developing and aging worms, respectively. We also found transcript effects linked to genotype [15.3% (2899) and 10.5% (1999) for developing and aging worms, respectively] and genotype-by-age effect [20.5% (3888) and 15.2% (2886)].

A GO enrichment analysis of the regulated genes (Table S3) showed genotypic differences in regulation of calcium transport and in structure molecules, such as collagen or extracellular matrix in developing worms. For aging worms, we found genotypic differences in regulation of response to oxidative stress and glycoprotein catabolic process. The genotype-by-age interaction affected signaling pathways mediated by cell surface receptors and metabolic processes for nitrogen, glutamine, glyoxylate, isocitrate, or malate in developing worms. In aging worms, regulated processes included cell division and lipid storage.

### Comparison of parental strains and RILs showed the polygenic nature of gene expression regulation in older worms

**Figure 1:**
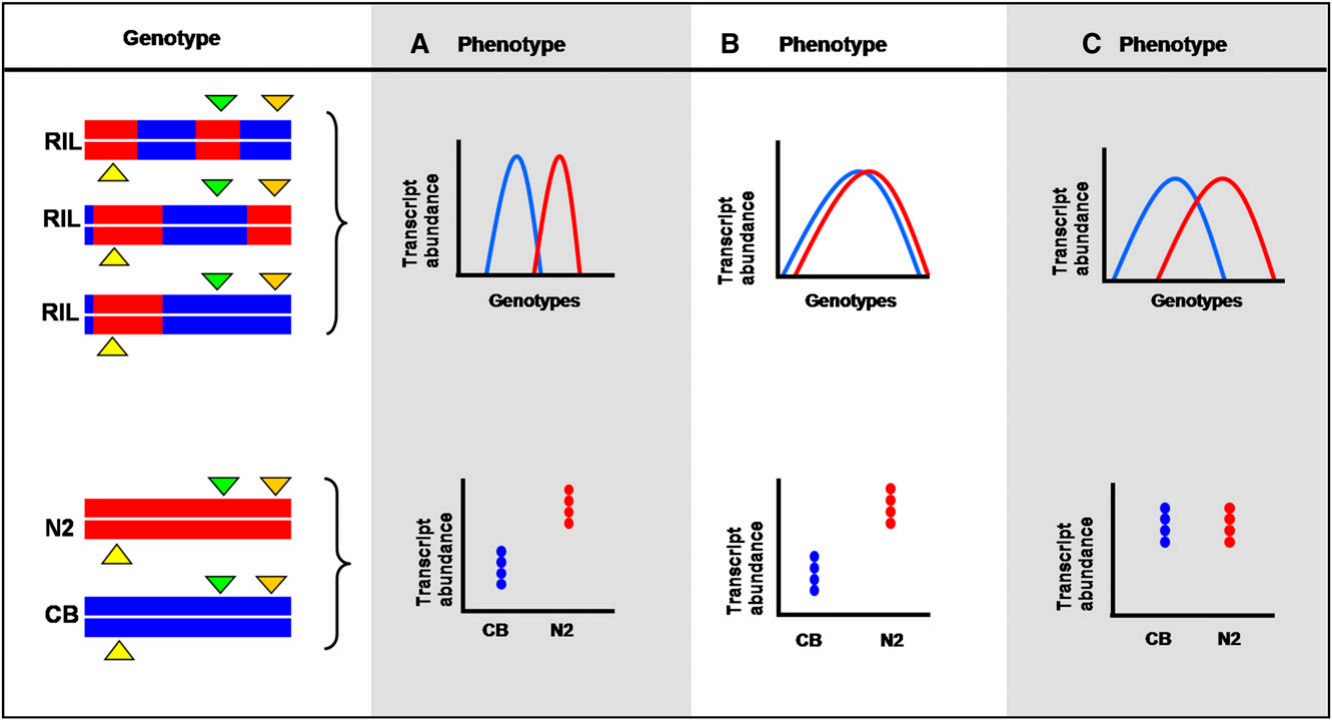
Interpretation of gene expression differences in segregating populations. Three different features could be identified when comparing mapping data from RILs and expression level differences between parental strains. (A) Genes with a statistical evidence for genomic linkage (eQTL) and different levels of transcript abundance between the parental lines. The regulatory elements (green and orange triangles) may have different effects (red or blue) over the phenotype (gene expression). Reshuffling the different regulatory elements in the RILs induced phenotypes in the population within the range of the parental strains (from red to blue). (B) Genes with no statistical evidence for genomic linkage (eQTL) and different levels of expression between the parental lines. The regulatory elements may have similar and/or opposite small effects. Their recombination induces intermediate phenotypes in the RILs. None of the single effects of any of the multiple regulators was large enough to be identified. Different mapping strategies or complementary experimental data may be able to identify multiple regulatory elements affecting the expression levels. (C) Genes with statistical evidence for genomic linkage (eQTL) and with similar levels of transcript abundance between the parental strains. The genomic recombination may have induced extreme phenotypes when compared to the parental lines. The expression level of some of those genes may show signs of transgressive segregation.

We investigated the overlap between differentially expressed genes for the parental strains and the genes for which we could identify an eQTL in the segregants. Figure 1 shows three possible relevant gene categories identified: (A) genes differentially expressed between the parental lines for which an eQTL was detected; (B) genes differentially expressed between the parental lines without an eQTL; and (C) genes not differentially expressed between the parental lines but with an eQTL.

Category A genes has a (partially) simple genetic architecture. These genes are differentially expressed between the parental lines and segregate, most likely as the result of a single and detectable strong effect locus. We found 496 genes in developing worms with a genotypic effect and 266 in aging worms. GO enrichment analysis (Table S4) showed ATPase activity, calcium transport, and protein kinase activity as strongly regulated processes in developing worms. In aging worms, we found genotypic effect for response to oxidative stress responses, metal ion binding, and hydrolase. Similarly we found 342 genes in developing worms with a genotype-by-age effect and 288 in aging worms (Table S1 and Figure S1, Figure S2, Figure S3, and Figure S4). Genes with a genotype-by-age effect in aging worms were related to oxidative stress responses and to metabolism of glutamine and glycoproteins and ATP-binding (Table S4).

In category B (Figure 1B), we identified 2535 genes (in developing worms) and 1803 genes (in aging worms) with a genotypic effect but without an eQTL (Table 1). For genotype-by-age interaction, 3650 genes (in developing worms) and 2741 genes (in aging worms) were found to be differentially expressed between the parental strains but without a corresponding _g×a_eQTL (genotype-by-age eQTL). For both eQTL and _g×a_eQTL, the failure in detection might be explained by multiple loci with small effects, in which none of these loci had a regulatory effect strong enough to be detected in the RILs.

**Table 1:** Number of genes overlapping between differentially expressed genes in the parental strains and genes for which an eQTL was mapped

	Developing			Aging	
	Genotype	Age*Genotype		Genotype	Age*Genotype
Genes with a significant eQTL	1401	935		867	844
Differentially expressed in the parents	2899	3888		1999	2886
Detected eQTL and differentially expressed in the parents	496	342		266	288
NO detected eQTL and differentially expressed in the parents	2535	3650		1803	2741
Detected eQTL and NOT differentially expressed in the parents	1041	691		673	688

The first two rows show the number of genes with an eQTL in the RILs and differentially expressed in the parental strains with genotype and age-by-genotype effects. The next three rows show the number of genes within the three categories considered: category A, genes with at least an eQTL and differentially expressed between the parents; category B, genes without an eQTL but differentially expressed in the parents; and category C, genes with an eQTL but not differentially expressed in the parents. Thresholds for eQTL detection: –log10 *P*-value > 3 and –log10 *P*-value > 2 for parental analysis.

In category C (Figure 1C), we found 1041 genes (in developing worms) and 673 genes (in aging worms) with an eQTL but no difference in expression between the parental strains. For the _g×a_eQTL, an equal number was found for developing (691) and aging (688) worms (Table 1). The eQTL in category C may be the result of new epistatic interactions in the RILs or complementary additive effects of the new allelic combinations (Rieseberg *et al.* 1999; Brachi *et al.* 2010). Both possibilities involve multiple regulatory elements and are likely to show transgression [*cf*. Gutteling *et al.* (2007a, b)] of gene expression due to higher or lower transcript abundance in a specific group of RILs than in either parent.

Compared with category A (genes differentially expressed between the parental lines for which an eQTL was detected), many more genes fall in category B (genes differentially expressed between the parental lines without an eQTL), suggesting the polygenic nature of gene expression regulation and the decreased activity of strong effect locus in older worms (Table 1). The difference between genes in category A and C (genes not differentially expressed between the parental lines but with an eQTL) suggested transgressive segregation of gene expression.

### Heritability of gene expression and eQTL changed with age

Failure to detect eQTL in genes differentially expressed between the parental lines (category B genes) suggested polygenic regulation of gene expression and activity of small effect regulatory locus. Furthermore, genes with high heritability values for transcript abundance (from now on, highly heritable genes) are more likely to have a detectable eQTL, and highly heritable genes without an eQTL suggest more polygenic regulation of gene expression. Therefore, we investigated how heritability (H^2^) of gene expression changes with age and its possible relation with the ability to detect eQTL in older worms.

**Figure 2:**
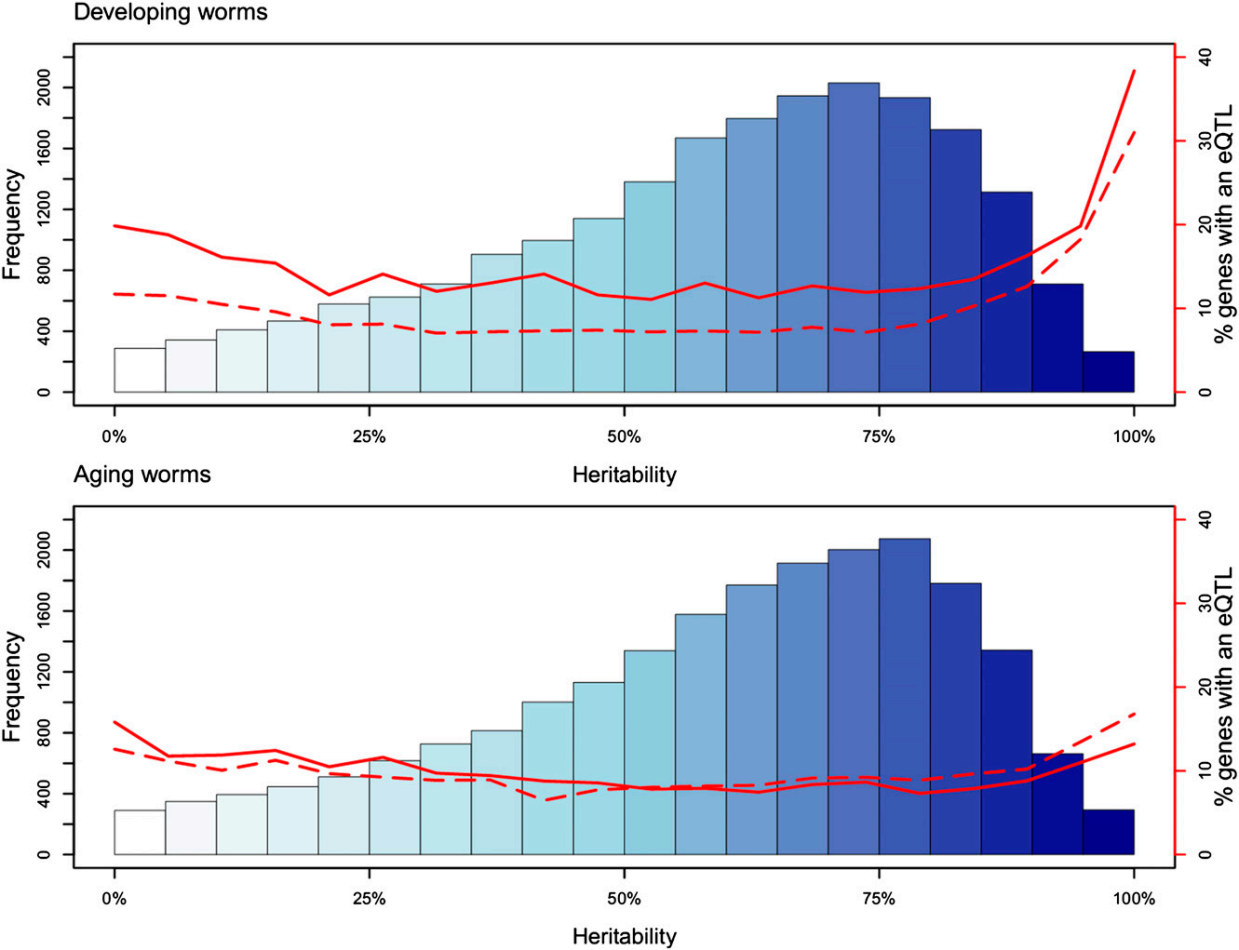
Heritability and eQTL. Heritability of gene expression in developing and aging worms was plotted. The y-axis (left side) shows the frequency of transcripts within a range of heritability values. The x-axis shows the percentage of heritability per gene. The red line (y-axis, right side) indicates the number of genes with an eQTL (solid line) and number of genes with an _a×g_eQTL (dashed line) within the range of heritability.

Heritability is the fraction of variation, in gene expression, that can be attributed to genotypic variation in the segregants. The heritability of each transcript was estimated from the pooled within line variance of the parents as in Keurentjes *et al.* (2007) (Figure 2). The mean heritability for developing worms was 0.64 and for aging worms 0.67. As expected (Brem *et al.* 2005), we observed that the percentage of genes for which an eQTL could be found increased with H^2^ for both young and developing worms. However, for old and for aging worms (reproductive to old worms), the percentage of genes for which an eQTL could be found hardly increased with an increasing H^2^ (Figure 2). Some of the highly heritable functions identified only in aging worms were gluconeogenesis, calcium channel activity, cholesterol binding, and protein kinase activities (complete GO analysis of those genes can be found in Table S5 and Table S6).

### Patterns of gene expression showed signs of transgressive segregation

To detect transgressive segregation, we compared expression levels of the parental and RILs in juvenile, reproductive, and old worms. We found 8205 (43.4%), 9109 (48.2%), and 8484 (44.9%) genes, respectively, in which expression levels transgressed. That is, per gene, six or more RILs had expression levels higher or lower than two times the standard deviation (2*SD) of either parent. This large transgression abundance in gene expression could be caused by the difference in physiological age between and among the RILs and parental lines. To get a better estimate of transgression, we investigated whether changes in gene expression would also transgress with age. Namely, a gene showed signs of transgressive segregation if six or more RILs had expression levels higher or lower than 2*SD of either parent in t1 and t2 (developing worms) or in t2 and t3 (aging worms). In this way, we identified 1032 genes (5.4%) with significant transgression of expression changes in developing worms, and 1122 genes (5.9%) in aging worms. The results show that transgressive segregation of gene expression was widespread, which is an indication of polygenic regulation of gene expression; however, transgression did not change with age.

### Heritability, number of eQTL, and transgressive segregation all pointed to increased polygenic regulation in old worms

**Figure 3:**
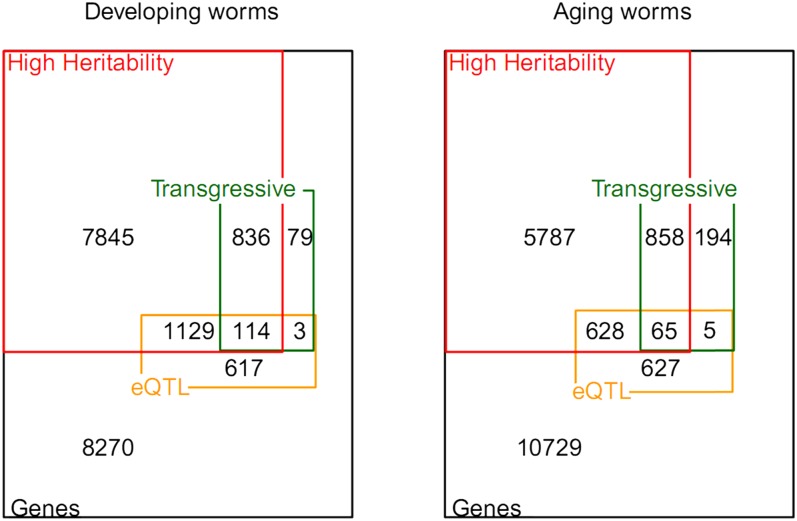
Overlapping features analyzed in developing and aging worms. From almost 19,000 analyzed genes (black square), we identified highly heritable genes (red square), genes with at least one detectable genomic linkage (eQTL, orange square), and genes in which expression values transgressed from the parental values (Transgressive, green square).

We identified highly heritable patterns of gene expression for developing worms (9924 genes with H^2^ > 0.69) and for aging worms (7338 genes with H^2^ > 0.77) (see *Materials and Methods* for threshold determination). The overlap between highly heritable genes, number of eQTL, and transgressive genes in developing and aging worms is shown in Figure 3. As expected from the comparison between eQTL and differentially expressed genes in the parental lines (Table 1 and Figure 3), the highly heritable genes were enriched for eQTL, especially for genes with H^2^ > 0.9 (Figure 2). Of the total genes with eQTL, we found 66.3% (developing) and 52.4% (aging) in the high heritability group. This finding shows heritability as a predictor of eQTL detection; however, it is not perfect. For instance, when we selected for high heritability before mapping, many eQTL would be missed because 33.7% (620) and 47.6% (632) of the genes with eQTL in developing and aging worms did not fall into the high heritability groups (see also Figure 2). The relative increase of genes with an eQTL but low heritability in aging worms indicates that age affects the relationship between heritability and the ability to detect eQTL. This age effect was also observed between heritability and transgression. In developing worms, 7.2% (82) of the genes showed transgression but did not have high heritability values, whereas in aging worms this increased to 17.7% (199) of the genes.

Genes with transgression but low heritability are interesting as they imply that the RILs have a low among-strain variance but are somehow shifted in mean value from the mid parent value. It implies that the transgression is due to heritable factors that do not contribute to the estimated heritability (*e.g.* epistatic interactions). Table S7 and Table S8 list those genes and a GO enrichment analysis. Interestingly, biological functions associated with those genes in developing worms (7.2%) were related to embryonic development and growth. On the other hand, in aging worms (17.7%), biological functions were related to transcription, signal transduction, and proteolysis. Nevertheless, in general, genes showing transgressive segregation have a higher heritability and are enriched with eQTL (Figure S5).

### The number of polymorphic regulators increased with age

**Figure 4:**
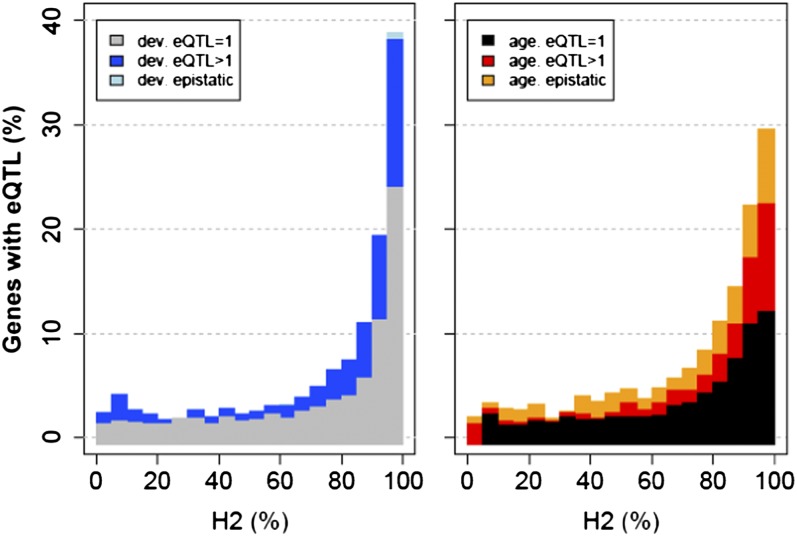
Percentage of eQTL (y-axis) detected in transcripts ordered by heritability (H2; x-axis). Developing worms are represented in the left panel and aging worms in the right panel. Total height of the bar indicates the percentage of genes for which eQTL could be found. Gray and black areas indicate the percentage of genes for which only one eQTL could be detected. Blue and light blue (for developing worms) and red and orange (for aging worms) areas show the percentage of genes with two or more eQTL. Light blue and orange areas indicate the percentage of genes for which signs of epistatic interactions were found. The relative increase in genes with polygenic regulation is largest in the highly heritable group of genes in aging worms.

Failure to detect eQTL may be explained by multiple regulatory elements in which effects cannot be detected using single marker analysis. Moreover, the differences in number of genes with eQTL, signs of transgression, and high heritability between developing and aging worms indicate that the power of eQTL detection was affected by age. To test this hypothesis, we applied a forward and backward marker selection approach on model 1 previously used for eQTL detection. The procedure selects per gene the markers and their interactions for the linear model 1 that would best explain the variation in expression. This strategy allowed identifying genes that were likely to have more than one polymorphic regulatory element affecting their expression values at different ages. A summary of the results is shown in Figure 4, where the number of transcripts was ordered by heritability classes (x-axis) and the percentage of detected eQTL per type was plotted (y-axis). In developing worms, we found that the number of transcripts with an eQTL increased when two or more markers were fitted into the model. This increment was larger for transcripts with higher heritability. Strikingly, in aging worms, the increase in eQTL was larger when two or more markers in the model were considered. Moreover, in developing worms, hardly any signs of epistatic interaction were found, whereas in aging worms, epistatic interaction made up a large part of the detectable genetic effects. This further strengthens the observation that heritable regulation of gene expression becomes more polygenic in aging worms due to involvement of multiple loci affecting the power of eQTL detection in older individuals.

## Discussion

Heritability provides a measurement of the phenotypic variation that can be attributed to genotypic variation and is an indicator of the relative importance of genes and environment in traits variation (Visscher *et al.* 2008). Understanding how heritability changes with age is important not only for eQTL mapping but also because age-specific heritability determines how populations respond to natural selection. This has been studied for life-history traits in cattle (Albuquerque and Meyer 2005), in fruit flies (Snoke and Promislow 2003), and in natural populations of swans (Charmantier *et al.* 2006). For gene expression, as for other traits, the power to detect loci that affect gene expression (eQTL) depends largely, but not exclusively, on heritability. We found a mean heritability of 0.67 in aging worms, which was not significantly different from developing worms (0.64). These findings are comparable to mean heritability values found for gene expression in RIL populations of yeast (Brem *et al.* 2005; Zou *et al.* 2009) and *Arabidopsis* (Keurentjes *et al.* 2007). But heritability of many phenotypic traits changes with age. For example, Rovers *et al.* (2002) found that heritability changed from 0.44 to 0.71 in a three-year study for otitis media (inflammation of the middle ear) in humans. Also, studies in cattle have reported heritability changes for morphological characteristics with age (Réale *et al.* 1999). Likewise, we found that for individual genes the heritability of gene expression between developing and aging worms was different, and therefore, the biological functions identified as highly heritable at different ages changed.

Our study also showed that heritability of gene transcript abundance and the number of eQTL are uncoupled in aging worms because of the increased polygenic nature of gene expression regulation. We found that with a single marker approach almost 20% of genes with a heritability > 0.9 had an eQTL in developing worms. Surprisingly, only 10% was found in old worms. Using a multimarker approach, this percentage increased to almost 30% for both age groups.

Recently, we showed that the number of detected heritable gene expression patterns declined with age (Viñuela *et al.* 2010a). Linkage analysis indicated that the decline in number of eQTL in older worms was stronger in *cis*-acting linkages than in *trans*-acting. In line with those results, our data presented here indicate that a major part of gene expression regulation becomes more polygenic in aging worms. This suggests that multiple intermediates involved in *trans*-acting regulation become relatively more influential with age. But care should be taken in making strong conclusions. The efficiency to map eQTL depends to a large extent on the *P*-value thresholds at which eQTL are mapped. This especially applies to *trans*-acting eQTL, because they exhibit smaller genetic effects (Petretto *et al.* 2006). Their smaller effect has been related to the number of protein intermediates (Smith and Kruglyak 2008). Polymorphic variation may be diluted over a larger number of proteins and, therefore, are more difficult to map. Moreover, *trans*-regulation can have intermediate regulators with opposing effects on transcripts levels. Such opposite effects can cancel each other in a large group of RILs, limiting the ability to detect genetic linkage (Brem *et al.* 2005; Smith and Kruglyak 2008). Therefore, it is likely that the relationship between heritability and eQTL detection was uncoupled in aging worms as a consequence of the increased polygenic and less strong gene expression regulation.

Transgressive segregation analysis and the comparison of RILs with parental strains suggest that gene expression regulation becomes more polygenic with age. We identified a similar number of genes in both age groups. Transgressive segregation is attributed to epistatic interactions between alleles or to opposite additive effects of segregating alleles (Rieseberg *et al.* 1999). Therefore, the high heritability of transgressive genes was expected. However, the number of transgressive genes for which we were able to identify genomic linkage was lower in aging worms, suggesting that a more complex polygenic regulation affected our ability to detect linkages at these ages. Genes with transgression but without high heritability suggests that the RILs do not have a high among-strain variance but are, to a certain extent, shifted in mean value from the mid parent value. This implies that the transgression is due to heritable factors that do not contribute to the estimated heritability, mainly due to epistatic interactions. To further refine these studies and focus on specific loci, introgression lines (Doroszuk *et al.* 2008; Kammenga *et al.* 2008) or a combination of RNAi knockdown experiments across different RILs (Elvin *et al.* 2011) can be used.

Stochasticity in gene expression is widely assumed to play a role in the aging process, and it may affect our ability to detect eQTL in aging worms. Bahar *et al.* (2006) reported in aging mouse heart an increased cell-to-cell variation in gene expression, and Herndon *et al.* (2002) reported muscle-specific decline in aging *C. elegans* associated with stochastic events. The latter study, however, also reported a well-maintained nervous system in senescent worms with no association with any stochastic event, suggesting a specific tissue aging rate. Genome-wide variation of gene expression increased in aging *C. elegans* worms (Viñuela *et al.* 2010a); however, some genes in aging worms were found to be strongly regulated and therefore had low levels of stochasticity. Those regulated genes in older individuals were associated with longevity, just as many other studies have identified regulated genes in older individuals (Golden and Melov 2004; Mccarroll *et al.* 2004). The question can be raised whether stochastic variation associated with aging is another factor explaining the increased discrepancy between eQTL and heritability values in older worms. The different number of highly heritable transcripts at both ages (9924 genes with H^2^ > 0.69 in developing worms, and 7338 genes with H^2^ > 0.77 in aging worms) could be due to increased noise in older worms. Because heritability is a measurement of the phenotypic variation that can be attributed to genotypic variation, it is assumed that the remaining variation is due to environmental changes. Here, aging might be considered an (internal) environmental factor. Changes in the environment (age) could induce phenotypic variation by means of stochasticity and therefore lead to lower heritability values and less highly heritable transcripts in older worms. However, an underestimation of heritability due to stochasticity does not explain the similar percentage of highly heritable genes for which we could not identify an eQTL (79% and 78.8%, respectively).Heritability of gene transcript abundance and the number of eQTL are uncoupled in aging worms because of the increased polygenic nature of gene expression regulation. We showed that heritability of gene expression changes with age. The mean heritability was similar for both age groups but not at the single gene level, where it changes with age. A possible reason could be age-dependent changes in gene expression regulation. A scenario in which regulation becomes more polygenic in aging worms would explain a large part of the decrease in eQTL. Likewise, it would explain the imbalance between highly heritable genes and eQTL in aging worms, as well as the smaller number of detected transgressive genes. These results might be important for understanding the failure to detect heritable variants in GWAS studies. As GWAS are enriched for eQTL, our results imply that GWAS should take into account the diminished effects of loci at older ages.

## Supplementary Material

Supporting Information
