# Supplementary material for: Aging Uncouples Heritability and Expression-QTL in Caenorhabditis elegans
Source: G3 (Bethesda). 2012 May 1;2(5):597–605. doi: 10.1534/g3.112.002212 (PMC3362942; doi:10.1534/g3.112.002212)
Supplement: Supporting Information [file supp_2_5_597__index.html]

Supporting Information 

# Aging Uncouples Heritability and Expression-QTL in *Caenorhabditis elegans*

## Supporting Information for Vinuela *et al*, 2012

**Files in this Data Supplement:**

- Supporting Information - Figures S1-S5 and Tables S1-S8 (PDF, 551 KB)
- Figure S1 - Number of overlapping genes between differentially expressed genes in the parental strains and with at least an eQTL at different thresholds in developing worms with genotype effect (PDF, 86 KB)
- Figure S2 - Number of overlapping genes between differentially expressed genes in the parental strains and with at least an eQTL at different thresholds in developing worms with genotype\*age interacting effect (PDF, 84 KB)
- Figure S3 - Number of overlapping genes between differentially expressed genes in the parental strains and with at least an eQTL at different thresholds in old worms with genotype effect (PDF, 84 KB)
- Figure S4 - Number of overlapping genes between differentially expressed genes in the parental strains and with at least an eQTL at different thresholds in old worms with genotype\*age interacting effect (PDF, 84 KB)
- Figure S5 - Transgressive segregation, heritability and eQTLs (PDF, 358 KB)
- Table S6 - Table summary with GO terms enrichment analysis summary for highly heritable genes in developing and aging worms (PDF, 107 KB)
- Table S1 - List of differentially expressed genes between N2 and CB4856 in juvenile (t1), reproductive (t2) and old worms (t3) (.xls, 685 KB)
- Table S2 - List of differentially expressed genes between N2 and CB4856 in developing (t1 vs. t2) and aging worms (t2 vs. t3) (.xls, 1.1 MB)
- Table S3 - Significantly enriched Gene Ontology (GO) terms and functional domains (.xls, 33 KB)
- Table S4 - Table with GO enrichment analysis of overlapping genes between differentially expressed genes in the parental strains with at least an eQTL in developing and aging worms (.xls, 25 KB)
- Table S5 - Table with GO enrichment analysis of highly heritable genes in developing and aging worms (.xls, 30 KB)
- Table S7 - Excel file with genes showing signs of transgressive segregation, but without high heritability in developing worms in one spreadsheet (.xls, 40 KB)
- Table S8 - Excel file with genes showing signs of transgressive segregation, but without high heritability in aging worms in one spreadsheet (.xls, 60 KB)
