# Supplementary material for: Aging Uncouples Heritability and Expression-QTL in Caenorhabditis elegans
Source: G3 (Bethesda). 2012 May 1;2(5):597–605. doi: 10.1534/g3.112.002212 (PMC3362942; doi:10.1534/g3.112.002212)
Supplement: Supporting Information [file supp_2.5.597_002212SI.pdf]

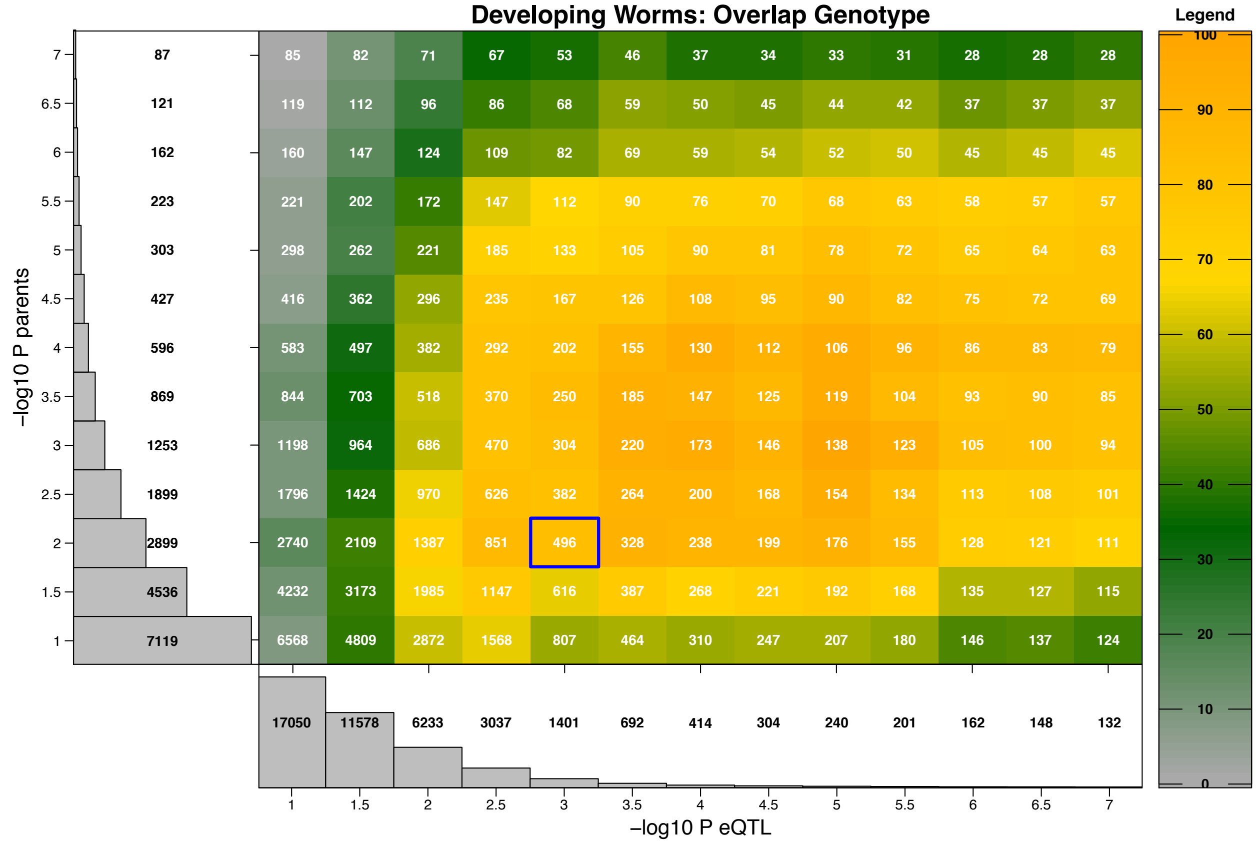

Figure S1 Number of overlapping genes between differentially expressed genes in the parental strains and with at least an eQTL at different thresholds in developing worms with genotype effect. *Bottom bar graphs* show number of genes with at least an eQTL at different thresholds (from –log10 P-value =1 to –log10 P-value = 7). *Left bar graphs* show number of differentially expressed genes between parental strains at different thresholds. The number of overlapping genes is shown in the *center graph*. The dark square indicates the number of overlapping genes at the joined-FDR (–log10 P-value =3 for eQTL analysis, –log10 P-value = 2 for parental strains analysis). A hypergeometric test was used to determine significance of the overlapping genes. The color legends for the significance are shown at the *Right graphs.*

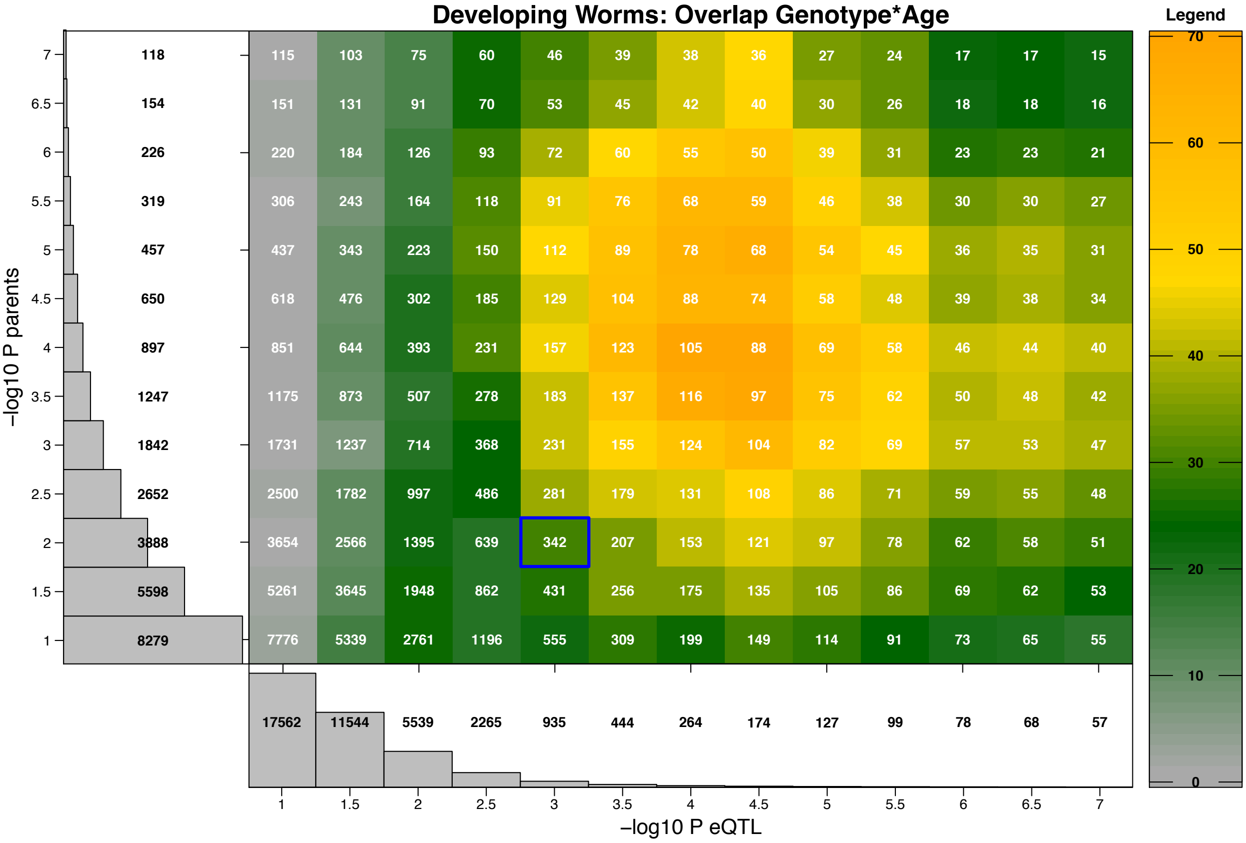

Figure S2 Number of overlapping genes between differentially expressed genes in the parental strains and with at least an eQTL at different thresholds in developing worms with genotype*age interacting effect. See Figure S1 legend for details.

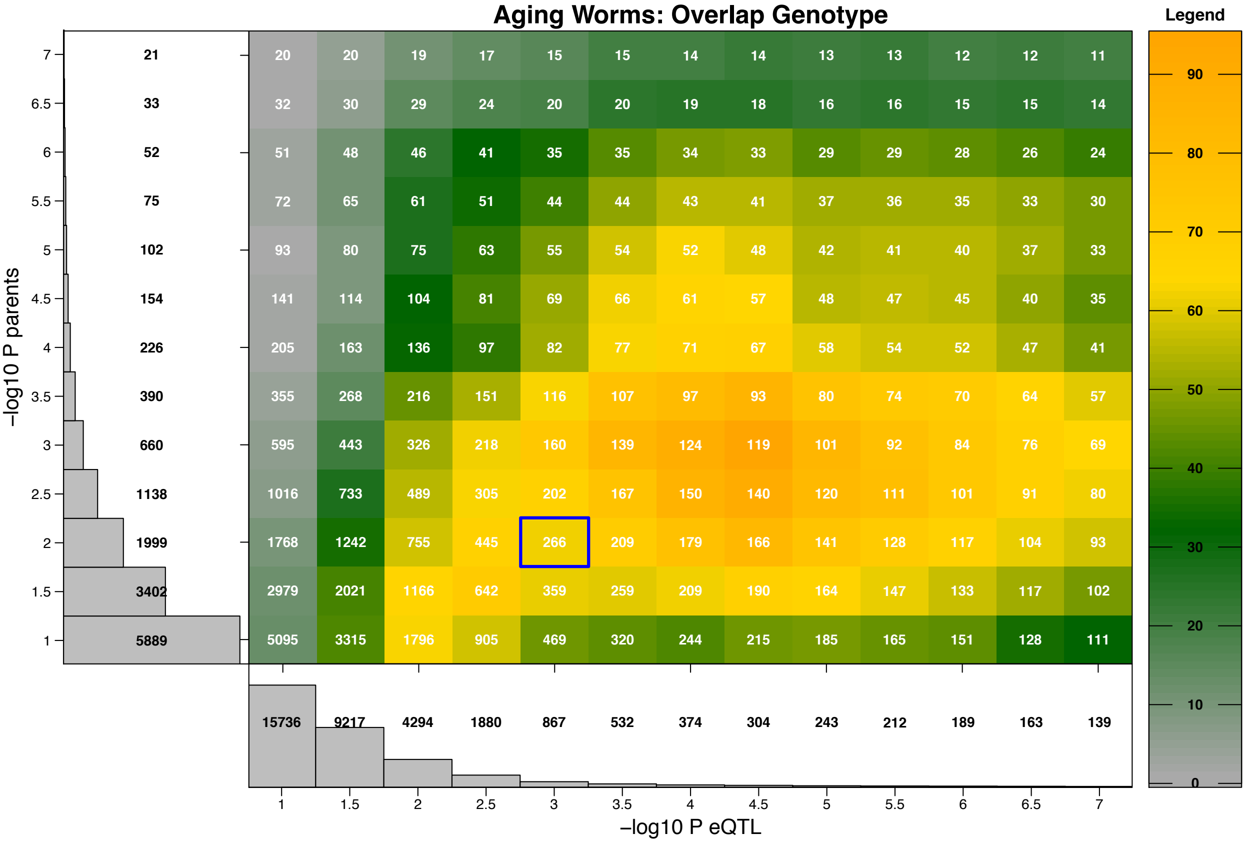

Figure S3 Number of overlapping genes between differentially expressed genes in the parental strains and with at least an eQTL at different thresholds in old worms with genotype effect. See Figure S1 legend for details.

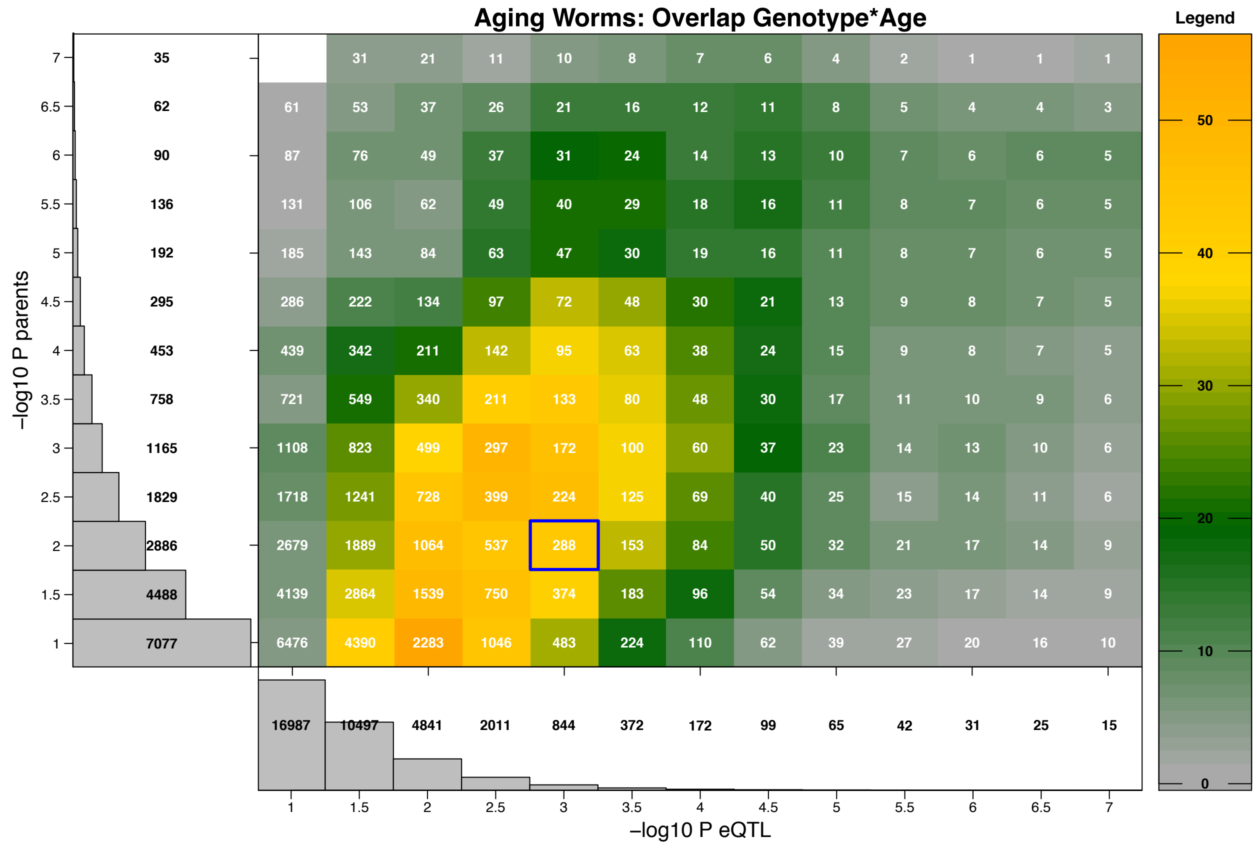

Figure S4 Number of overlapping genes between differentially expressed genes in the parental strains and with at least an eQTL at different thresholds in old worms with genotype*age interacting effect. See Figure S1 legend for details.

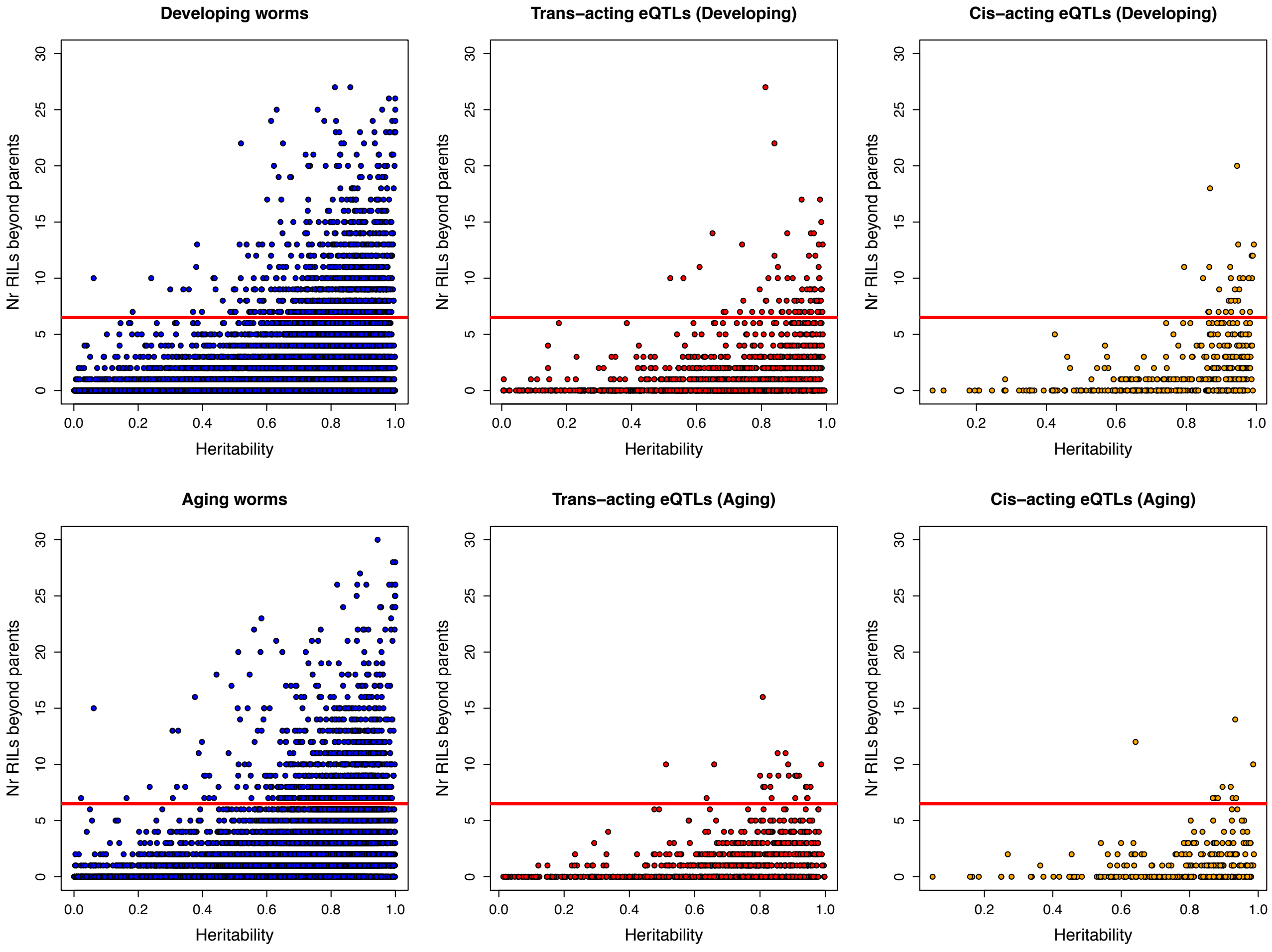

Figure S5 Transgressive segregation, heritability and eQTLs. Upper graphs show values for developing worms, lower graphs for aging worms. Left graphs (upper and lower) show the number of RILs which expression values transgress from the higher or lower parent value (*y-axis*) *versus* their heritability values (*x-axis*). Middle and right graphs plot the number of genes with eQTLs (*trans*-acting middle, *cis*-acting right) detected, their heritability values and their transgressive segregants. We detected more transgressive segregation in genes with *trans*-acting regulation and in developing worms than in aging worms and *cis*-acting eQTLs. Red line indicates the threshold for significant transgressive segregation.

Tables S1 –S8 are available for download at

http://www.g3journal.org/lookup/suppl/doi:10.1534/g3.112.002212/-/DC1.

Table S1 List of differentially expressed genes between N2 and CB4856 in juvenile (t1), reproductive (t2) and old worms (t3). Genes with a –log10 P-value > 2 in a linear model 1 (gene expression = marker (effect) + error) were considered significant. Array ID = identification number for microarray spot. CDS name = coding sequence name. Gene name = public gene name. P-value = -log10 P-value for the significant test.

Table S2 List of differentially expressed genes between N2 and CB4856 in developing (t1 vs. t2) and aging worms (t2 vs. t3). Genes with a –log10 P-value > 2 in a linear model 1 (gene expression = marker(effect) + physiological age + interaction (marker*physiological age) + error) were considered significant. *Array ID* = identification number for microarray spot. *CDS name* = coding sequence name. *Gene name* = public gene name. *P-value* = -log10 P-value for the significant test. Worksheets = *Developing (marker)* for genes differentially regulated by genotype in developing worms; *Developing (marker-age)* for genes differentially regulated by genotype*age interaction in developing worms; *Aging (marker)* for genes differentially regulated by genotype in aging worms; *Aging (marker-age)* for genes differentially regulated by genotype*age interaction in aging worms.

Table S3 Significantly enriched Gene Ontology (GO) terms and functional domains. *GO terms worksheets* contain full GO enriched analysis for differentially expressed genes between parental strains in developing and aging worms, genotype effect and genotype*age interacting effect. *GO terms* are the GO id numbers. Total genes are the total number of genes in *C. elegans* assigned to each GO term. *Number of genes* are the number of regulated genes by genotype or genotype*age in each GO term. *P-values* were calculated using hypergeometric test, values lower than 0.01 and more than two genes in a category were considered significant. The hypergeometric test compared a group of 2779 GO terms, with 18,312 annotated genes. Gene annotation with GO terms was extracted from Wormbase release WB210.

Table S4 Table with GO enrichment analysis of overlapping genes between differentially expressed genes in the parental strains with at least an eQTL in developing and aging worms.

Table S5 Table with GO enrichment analysis of highly heritable genes in developing and aging worms.

Table S6 Table summary with GO terms enrichment analysis summary for highly heritable genes in developing and aging worms. First table shows GO terms significantly enriched in both groups of genes with a P-value < 0.01. Second table shows GO terms significantly enriched (P-value < 0.01) in developing worm but not in aging worms. Third table shows GO terms significantly enriched (P-value < 0.01) in aging worms but not in developing worms.

Table S7 Excel file with genes showing signs of transgressive segregation, but without high heritability in developing worms in one spreadsheet. GO enrichment analysis of the genes is shown in the other spreadsheet.

Table S8 Excel file with genes showing signs of transgressive segregation, but without high heritability in aging worms in one spreadsheet. GO enrichment analysis of the genes is shown in the other spreadsheet.

Table S6 Table summary with GO terms enrichment analysis summary for highly heritable genes in developing and aging worms. First table shows GO terms significantly enriched in both groups of genes with a P-value < 0.01. Second table shows GO terms significantly enriched (P-value < 0.01) in developing worms genes but not in aging worms. Third table shows GO terms significantly enriched (P-value < 0.01) in aging worms genes but not in developing worms

Common Go Terms enriched in Developing and Aging worms highly heritable genes				
GO terms	Genes in GO	Genes	P-value	Description
GO:0004030	4	4	0	aldehyde dehydrogenase [NAD(P)+] activity
GO:0006081	4	4	0	cellular aldehyde metabolic process
GO:0016763	6	6	0	transferase activity, transferring pentosyl groups
GO:0051020	6	6	0	GTPase binding
GO:0042302	309	191	0.00266	structural constituent of cuticle

Go Terms enriched only in Developing worms highly heritable genes				
GO terms	Genes in GO	Genes	P-value	Description
GO:0000795	4	4	0	synaptonemal complex
GO:0006544	4	4	0	glycine metabolic process
GO:0007611	4	4	0	learning or memory
GO:0008176	4	4	0	tRNA (guanine-N7-)-methyltransferase activity
GO:0016328	4	4	0	lateral plasma membrane
GO:0016358	4	4	0	dendrite development
GO:0016471	4	4	0	vacuolar proton-transporting V-type ATPase complex
GO:0018738	4	4	0	S-formylglutathione hydrolase activity
GO:0045773	4	4	0	positive regulation of axon extension
GO:0060102	4	4	0	collagen and cuticulin-based cuticle extracellular matrix
GO:0000104	5	5	0	succinate dehydrogenase activity
GO:0005581	5	5	0	collagen
GO:0003896	6	6	0	DNA primase activity
GO:0006269	6	6	0	DNA replication, synthesis of RNA primer
GO:0030071	6	6	0	regulation of mitotic metaphase/anaphase transition
GO:0044249	6	6	0	cellular biosynthetic process
GO:0004926	9	9	0	non-G-protein coupled 7TM receptor activity
GO:0005744	8	7	0.007421	mitochondrial inner membrane presequence translocase complex
GO:0015085	8	7	0.007421	calcium ion transmembrane transporter activity
GO:0016538	8	7	0.007421	cyclin-dependent protein kinase regulator activity
GO:0032580	8	7	0.007421	Golgi cisterna membrane

GO:0005388	12	11	0.000639	calcium-transporting ATPase activity
GO:0005604	14	12	0.002407	basement membrane
GO:0000776	14	13	0.000187	kinetochore
GO:0004091	17	14	0.003357	carboxylesterase activity
GO:0008233	50	35	0.007561	peptidase activity
GO:0005198	297	181	0.007627	structural molecule activity
GO:0010171	734	428	0.009828	body morphogenesis

Go Terms enriched only in Aging worms highly heritable genes				
GO terms	Genes in GO	Genes	P-value	Description
GO:0015485	4	4	0	cholesterol binding
GO:0004657	6	5	0.004131	proline dehydrogenase activity
GO:0004768	5	5	0	stearoyl-CoA 9-desaturase activity
GO:0006562	6	5	0.004131	proline catabolic process
GO:0006937	6	5	0.004131	regulation of muscle contraction
GO:0010085	6	5	0.004131	polarity specification of proximal/distal axis
GO:0032504	5	5	0	multicellular organism reproduction
GO:0006537	8	6	0.008598	glutamate biosynthetic process
GO:0004611	8	7	0.000662	phosphoenolpyruvate carboxykinase activity
GO:0005882	8	7	0.000662	intermediate filament
GO:0006505	9	7	0.00384	GPI anchor metabolic process
GO:0017076	8	7	0.000662	purine nucleotide binding
GO:0043073	9	7	0.00384	germ cell nucleus
GO:0004368	10	8	0.001697	glycerol-3-phosphate dehydrogenase activity
GO:0009331	10	8	0.001697	glycerol-3-phosphate dehydrogenase complex
GO:0034607	11	8	0.005989	turning behavior involved in mating
GO:0004652	12	9	0.002843	polynucleotide adenylyltransferase activity
GO:0006072	13	9	0.007881	glycerol-3-phosphate metabolic process
GO:0043631	12	9	0.002843	RNA polyadenylation
GO:0005262	15	10	0.009457	calcium channel activity
GO:0006094	13	11	0.000139	gluconeogenesis
GO:0016831	25	16	0.004391	carboxy-lyase activity
GO:0031625	26	16	0.007977	ubiquitin protein ligase binding
GO:0000122	27	17	0.004688	negative regulation of transcription from RNA polymerase II promoter
GO:0004518	34	23	0.000305	nuclease activity
GO:0001703	74	40	0.005349	gastrulation with mouth forming first
GO:0004713	809	359	0.004876	protein tyrosine kinase activity

GO:0004672	902	403	0.001747	protein kinase activity
GO:0004674	958	427	0.001619	protein serine/threonine kinase activity
GO:0006468	1013	452	0.001099	protein amino acid phosphorylation
GO:0005524	2060	879	0.00497	ATP binding
GO:0009790	2334	992	0.004822	embryonic development
