# Supplementary material for: Aging Uncouples Heritability and Expression-QTL in Caenorhabditis elegans
Source: G3 (Bethesda). 2012 May 1;2(5):597–605. doi: 10.1534/g3.112.002212 (PMC3362942; doi:10.1534/g3.112.002212)
Supplement: Supporting Information [file supp_2.5.597_FigureS3.pdf]

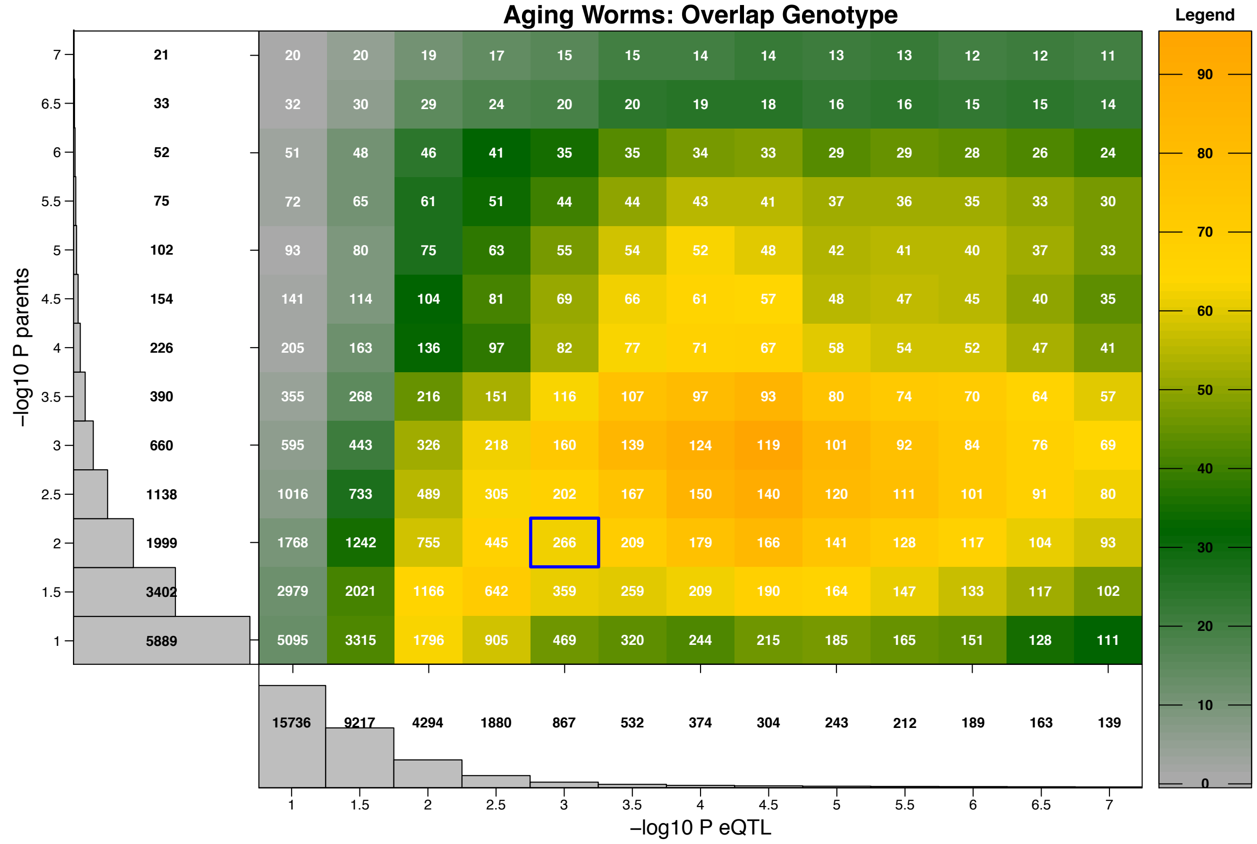

Figure S3 Number of overlapping genes between differentially expressed genes in the parental strains and with at least an eQTL at different thresholds in old worms with genotype effect. See Figure S1 legend for details.
