# Supplementary material for: Aging Uncouples Heritability and Expression-QTL in Caenorhabditis elegans
Source: G3 (Bethesda). 2012 May 1;2(5):597–605. doi: 10.1534/g3.112.002212 (PMC3362942; doi:10.1534/g3.112.002212)
Supplement: Supporting Information [file supp_2.5.597_FigureS4.pdf]

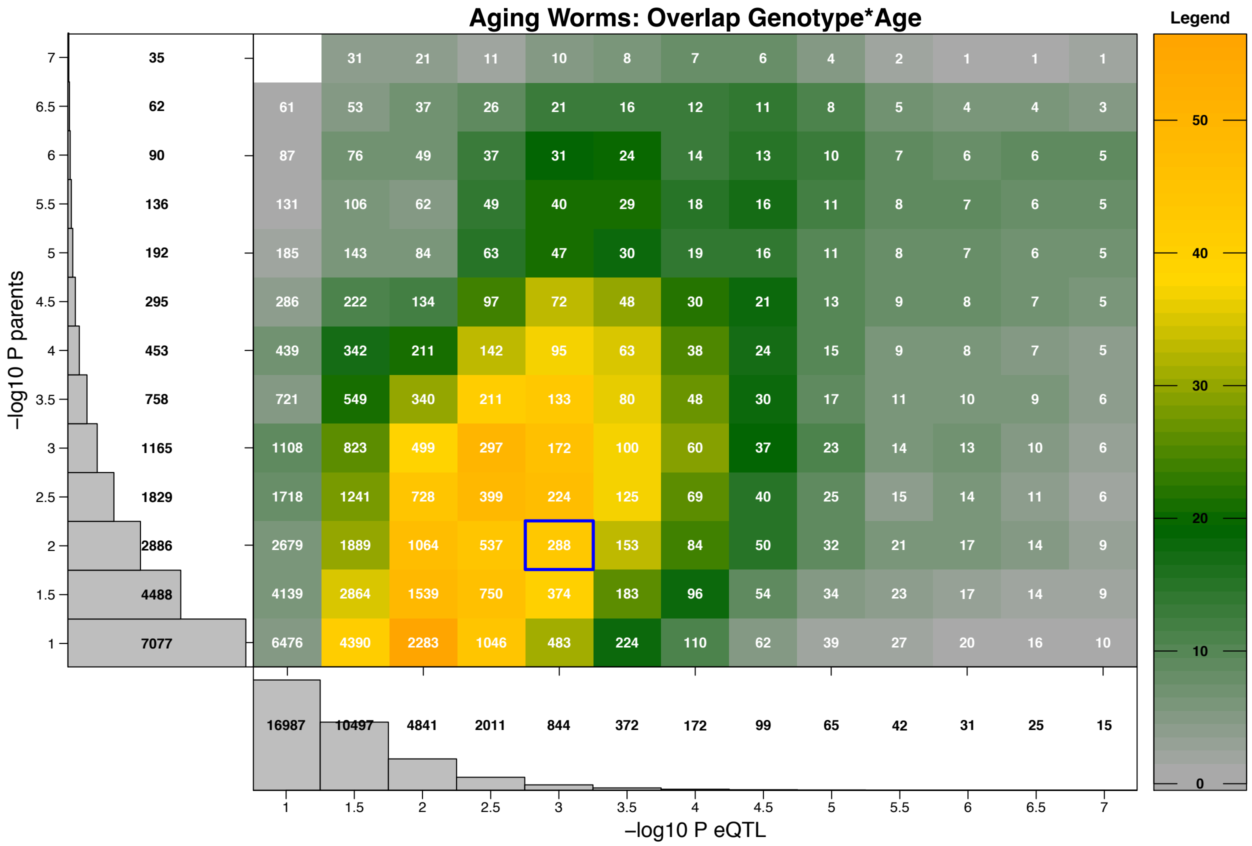

Figure S4 Number of overlapping genes between differentially expressed genes in the parental strains and with at least an eQTL at different thresholds in old worms with genotype*age interacting effect. See Figure S1 legend for details.
