# Supplementary material for: Aging Uncouples Heritability and Expression-QTL in Caenorhabditis elegans
Source: G3 (Bethesda). 2012 May 1;2(5):597–605. doi: 10.1534/g3.112.002212 (PMC3362942; doi:10.1534/g3.112.002212)
Supplement: Supporting Information [file supp_2.5.597_FigureS5.pdf]

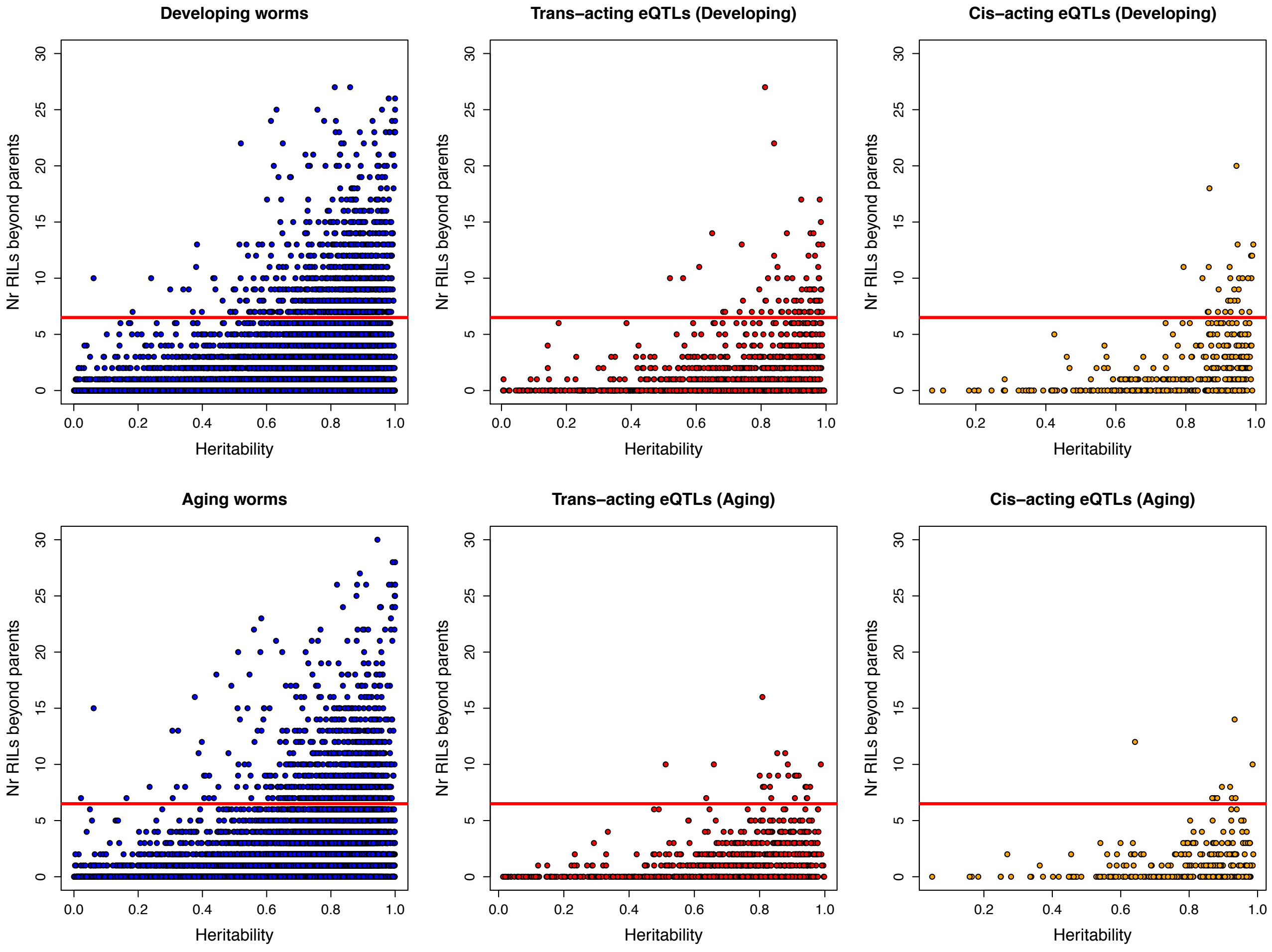

Figure S5 Transgressive segregation, heritability and eQTLs. Upper graphs show values for developing worms, lower graphs for aging worms. Left graphs (upper and lower) show the number of RILs which expression values transgress from the higher or lower parent value (*y-axis*) *versus* their heritability values (*x-axis*). Middle and right graphs plot the number of genes with eQTLs (*trans*-acting middle, *cis*-acting right) detected, their heritability values and their transgressive segregants. We detected more transgressive segregation in genes with *trans*-acting regulation and in developing worms than in aging worms and *cis*-acting eQTLs. Red line indicates the threshold for significant transgressive segregation.
