# Supplementary material for: Aging Uncouples Heritability and Expression-QTL in Caenorhabditis elegans
Source: G3 (Bethesda). 2012 May 1;2(5):597–605. doi: 10.1534/g3.112.002212 (PMC3362942; doi:10.1534/g3.112.002212)
Supplement: Supporting Information [file supp_2.5.597_TableS6.pdf]

**Table S6 Table summary with GO terms enrichment analysis summary for highly heritable genes in developing and aging worms.** First table shows GO terms significantly enriched in both groups of genes with a P-value < 0.01. Second table shows GO terms significantly enriched (P-value < 0.01) in developing worms genes but not in aging worms. Third table shows GO terms significantly enriched (P-value < 0.01) in aging worms genes but not in developing worms

| Common Go Terms enriched in Developing and Aging worms highly heritable genes | | | | |
|---|---|---|---|---|
| GO terms | Genes in GO | Genes | P-value | Description |
| GO:0004030 | 4 | 4 | 0 | aldehyde dehydrogenase [NAD(P)+] activity |
| GO:0006081 | 4 | 4 | 0 | cellular aldehyde metabolic process |
| GO:0016763 | 6 | 6 | 0 | transferase activity, transferring pentosyl groups |
| GO:0051020 | 6 | 6 | 0 | GTPase binding |
| GO:0042302 | 309 | 191 | 0.00266 | structural constituent of cuticle |

| Go Terms enriched only in Developing worms highly heritable genes | | | | |
|---|---|---|---|---|
| GO terms | Genes in GO | Genes | P-value | Description |
| GO:0000795 | 4 | 4 | 0 | synaptonemal complex |
| GO:0006544 | 4 | 4 | 0 | glycine metabolic process |
| GO:0007611 | 4 | 4 | 0 | learning or memory |
| GO:0008176 | 4 | 4 | 0 | tRNA (guanine-N7-)-methyltransferase activity |
| GO:0016328 | 4 | 4 | 0 | lateral plasma membrane |
| GO:0016358 | 4 | 4 | 0 | dendrite development |
| GO:0016471 | 4 | 4 | 0 | vacuolar proton-transporting V-type ATPase complex |
| GO:0018738 | 4 | 4 | 0 | S-formylglutathione hydrolase activity |
| GO:0045773 | 4 | 4 | 0 | positive regulation of axon extension |
| GO:0060102 | 4 | 4 | 0 | collagen and cuticulin-based cuticle extracellular matrix |
| GO:0000104 | 5 | 5 | 0 | succinate dehydrogenase activity |
| GO:0005581 | 5 | 5 | 0 | collagen |
| GO:0003896 | 6 | 6 | 0 | DNA primase activity |
| GO:0006269 | 6 | 6 | 0 | DNA replication, synthesis of RNA primer |
| GO:0030071 | 6 | 6 | 0 | regulation of mitotic metaphase/anaphase transition |
| GO:0044249 | 6 | 6 | 0 | cellular biosynthetic process |
| GO:0004926 | 9 | 9 | 0 | non-G-protein coupled 7TM receptor activity |
| GO:0005744 | 8 | 7 | 0.007421 | mitochondrial inner membrane presequence translocase complex |
| GO:0015085 | 8 | 7 | 0.007421 | calcium ion transmembrane transporter activity |
| GO:0016538 | 8 | 7 | 0.007421 | cyclin-dependent protein kinase regulator activity |
| GO:0032580 | 8 | 7 | 0.007421 | Golgi cisterna membrane |

| | | | | |
|---|---|---|---|---|
| GO:0005388 | 12 | 11 | 0.000639 | calcium-transporting ATPase activity |
| GO:0005604 | 14 | 12 | 0.002407 | basement membrane |
| GO:0000776 | 14 | 13 | 0.000187 | kinetochore |
| GO:0004091 | 17 | 14 | 0.003357 | carboxylesterase activity |
| GO:0008233 | 50 | 35 | 0.007561 | peptidase activity |
| GO:0005198 | 297 | 181 | 0.007627 | structural molecule activity |
| GO:0010171 | 734 | 428 | 0.009828 | body morphogenesis |

Go Terms enriched only in Aging worms highly heritable genes

| GO terms | Genes in GO | Genes | P-value | Description |
|---|---|---|---|---|
| GO:0015485 | 4 | 4 | 0 | cholesterol binding |
| GO:0004657 | 6 | 5 | 0.004131 | proline dehydrogenase activity |
| GO:0004768 | 5 | 5 | 0 | stearoyl-CoA 9-desaturase activity |
| GO:0006562 | 6 | 5 | 0.004131 | proline catabolic process |
| GO:0006937 | 6 | 5 | 0.004131 | regulation of muscle contraction |
| GO:0010085 | 6 | 5 | 0.004131 | polarity specification of proximal/distal axis |
| GO:0032504 | 5 | 5 | 0 | multicellular organism reproduction |
| GO:0006537 | 8 | 6 | 0.008598 | glutamate biosynthetic process |
| GO:0004611 | 8 | 7 | 0.000662 | phosphoenolpyruvate carboxykinase activity |
| GO:0005882 | 8 | 7 | 0.000662 | intermediate filament |
| GO:0006505 | 9 | 7 | 0.00384 | GPI anchor metabolic process |
| GO:0017076 | 8 | 7 | 0.000662 | purine nucleotide binding |
| GO:0043073 | 9 | 7 | 0.00384 | germ cell nucleus |
| GO:0004368 | 10 | 8 | 0.001697 | glycerol-3-phosphate dehydrogenase activity |
| GO:0009331 | 10 | 8 | 0.001697 | glycerol-3-phosphate dehydrogenase complex |
| GO:0034607 | 11 | 8 | 0.005989 | turning behavior involved in mating |
| GO:0004652 | 12 | 9 | 0.002843 | polynucleotide adenylyltransferase activity |
| GO:0006072 | 13 | 9 | 0.007881 | glycerol-3-phosphate metabolic process |
| GO:0043631 | 12 | 9 | 0.002843 | RNA polyadenylation |
| GO:0005262 | 15 | 10 | 0.009457 | calcium channel activity |
| GO:0006094 | 13 | 11 | 0.000139 | gluconeogenesis |
| GO:0016831 | 25 | 16 | 0.004391 | carboxy-lyase activity |
| GO:0031625 | 26 | 16 | 0.007977 | ubiquitin protein ligase binding |
| GO:0000122 | 27 | 17 | 0.004688 | negative regulation of transcription from RNA polymerase II promoter |
| GO:0004518 | 34 | 23 | 0.000305 | nuclease activity |
| GO:0001703 | 74 | 40 | 0.005349 | gastrulation with mouth forming first |
| GO:0004713 | 809 | 359 | 0.004876 | protein tyrosine kinase activity |

| | | | | |
|---|---|---|---|---|
| GO:0004672 | 902 | 403 | 0.001747 | protein kinase activity |
| GO:0004674 | 958 | 427 | 0.001619 | protein serine/threonine kinase activity |
| GO:0006468 | 1013 | 452 | 0.001099 | protein amino acid phosphorylation |
| GO:0005524 | 2060 | 879 | 0.00497 | ATP binding |
| GO:0009790 | 2334 | 992 | 0.004822 | embryonic development |
